# Wild Populations of *Triatoma infestans* Are Highly Connected to Intra-Peridomestic Conspecific Populations in the Bolivian Andes

**DOI:** 10.1371/journal.pone.0080786

**Published:** 2013-11-20

**Authors:** Simone Frédérique Brenière, Renata Salas, Rosio Buitrago, Philippe Brémond, Victor Sosa, Marie-France Bosseno, Etienne Waleckx, Stéphanie Depickère, Christian Barnabé

**Affiliations:** 1 Health Department, MIVEGEC (Université de Montpellier 1 et 2, CNRS 5290, IRD 224), Maladies Infectieuses et Vecteurs : Ecologie, Génétique, Evolution et Contrôle, Institut de recherche pour le développement (IRD), Montpellier, France; 2 Instituto Nacional de Laboratorios de Salud (INLASA), Laboratorio de Entomología Médica, La Paz, Bolivia; 3 Instituto de Investigaciones Biomédicas y de Interacción Social (IIBISMED), Facultad de Medicina, Universidad Mayor de San Simón, Cochabamba, Bolivia; 4 Laboratorio de Parasitología, Centro de Investigaciones Regionales Dr. Hideyo Noguchi, Mérida, Yucatán, México; Universidade Federal do Rio de Janeiro, Brazil

## Abstract

*Triatoma infestans*, the major vector of Chagas disease south of the Amazon in South America, has a large distribution of wild populations, contrary to what has previously been stated. These populations have been suspected of being the source of reinfestation of human habitats and could impede the full success of vector control campaigns. This study examined gene flow between intra-peridomestic populations and wild populations collected in the surround areas in three Andean localities in Bolivia. The populations were defined according to temporal, ecological, and spatial criteria. After DNA extraction from the legs of each insect, the samples were analyzed using seven microsatellite markers. First, the analysis of molecular variance (AMOVA) detected an absence of differentiation between wild and intra-peridomestic populations, although strong structuring was observed between the populations within each environment. Then for some populations, the Bayesian method of assignment to inferred populations showed very similar assignment patterns of the members of wild or intra-peridomestic populations in each locality. Finally, the detection of the first-generation migrants within the different populations provided evidence of insect displacement from the wild to the intra-peridomestic environment. This result indicates that, after control campaigns in the Andes, controlling this new paradigm of vector transmission risk stemming from the invasion of human habitats by wild populations of *T. infestans* requires long-term maintenance of public monitoring to keep the risk at a minimal level. Since wild populations of *T. infestans* have also been detected elsewhere in Argentina, Paraguay, and Chile, there is an urgent need to take these populations into account in future monitoring of Chagas disease transmission.

## Introduction

In the Southern Cone countries of South America and in Bolivia, *Triatoma infestans* (Reduviidae, Triatominae) remains the main and most widespread vector of *Trypanosoma cruzi*, the causative agent of Chagas disease. This species has long been considered almost exclusively domestic, but the increase in wild population discoveries in the Andes, in the Gran Chaco lowland region, and in Chile shows that wild *T. infestans* populations are much more widespread than previously thought [1-7]; the potential map of the distribution of wild *T. infestans*, based on an earlier sample of Bolivian and Argentinean wild foci, supports a very wide distribution [8]. 

In 1991, the regional program (INCOSUR-Chagas) of the Southern Cone countries was initiated, aiming to control Chagas disease and eliminate *T. infestans* based on house spraying with residual insecticides. This program has led to the interruption of vector-borne transmissions to humans in Chile, Uruguay, Brazil, eastern Paraguay, and parts of Argentina [9,10]. However, *T. infestans* persists primarily in the Bolivian Andean valleys and in different locations of the Gran Chaco ecoregion. Interestingly, these occurrences match the distribution where wild populations have been found. Determining whether the success in eliminating vectors could be disrupted by repeated reinfestations of dwellings and peridomestic areas by the nearby wild populations remains an open question. In this case, the current vigilance system, based on selective insecticide spraying of dwellings where vectors are resurgent, remains of limited efficacy and new control strategies must be developed [4,11-13]. 

Studies in the Gran Chaco region have highlighted that the main source of reinfestation was from habitats within a village rather than from nearby villages [14-17], emphasizing the role of reemerging peridomestic populations as the primarily source of dispersion [18]. Nevertheless, studies of populations at capture sites within villages, using different genetic markers (allozymes, microsatellites, etc.), depicted a high degree of microgeographical genetic structure among populations in the Andean as well as Gran Chaco regions [16,19,20]; a substantial differentiation between populations, even separated by short distances (around 10 m), has been observed. In conclusion, the genetic structure of *T. infestans* in human habitats may be based on infrequent events of bug displacements between ecotopes, giving rise to reduced genetic flow, population isolation, and genetic drift at the local level (colony). Since the assumption of movement of *T. infestans* populations between the wilderness environment and human habitats is recent, few studies are available. Given that the reinfestation phenomenon has been recurring in the Argentinean Chaco for several years, researchers have strengthened the search for wild populations and discovered their existence. In order to assess the origin of reinfesting bugs, they have used several methods, including the analysis of genetic markers (microsatellites and mitochondrial genes), and for the first time assessed reinvasion of domestic and peridomestic structures by nearby wild *T. infestans* colonies [5]. 

As part of the “TiBo” project founded by the ANR (French Research National Agency), aimed to depict the epidemiological role of the wild *T. infestans* population in Bolivia, unexpectedly, several new sites of wild populations were discovered in the Inter-Andean Dry Forest and Prepuna Andean ecoregions [21]. These bugs exhibited very high infection rates by *T. cruzi*, up to 85.7% in adults captured in the Andean valleys of La Paz Department [4].

Insofar as (i) *T. infestans* is one of the triatomine species that is best adapted to human habitats, (ii) it is an efficient vector, (iii) there is no genetic argument that has questioned the specific or subspecific status of the wild populations, and (iv) their infection rate can be extremely high, the invasion of human habitats by wild Bolivian populations is an extreme danger, potentially leading to resurgence of transmission in areas were domestic populations of triatomines have been controlled.

To establish the population source of reinfesting bugs (residual specimens after the chemical spray of the houses or migrants of domestic or wild origin) observed in three Bolivian Andean areas (two rural and one urban), populations were defined at a small geographical scale (capture site level) according to their habitat (indoors, peridomestic structures, and natural structures found in agropastoral or wild areas), and genetic variation was examined at seven microsatellite DNA loci. Briefly, the results support the displacement of bugs from wild to intra-peridomestic ecotopes as well as displacements between sites with the same type of ecotope.

## Materials and Methods

The field work was realized in collaboration and coordination with the SEDES (Health Departmental Service) of the Bolivian La Paz and Cochabamba Departments, and with the agreement of local authorities in each location (see material and methods). Search for triatomines in private spaces was done with the consent of every inhabitant who accompanied the active search for triatomines by the team members. The field studies did not involve endangered or protected species.

### Geographical origin of *T. infestans*

**Figure 1:**
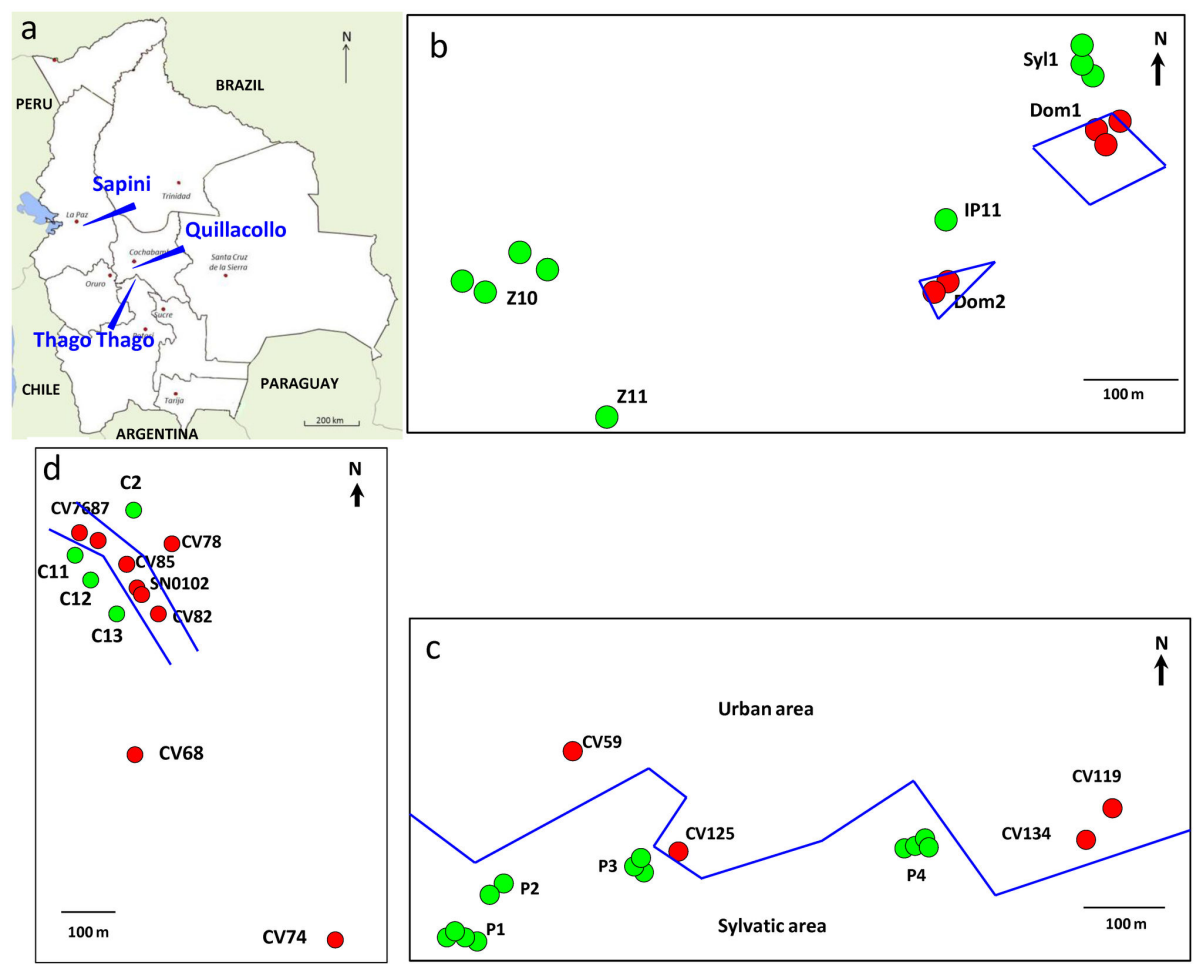
Geographical localization of wild and intra-peridomestic populations of *T. infestans*. a – General localization of the three study areas in Bolivia, blue arrows and names. b – Rural locality of Sapini in La Paz Department, inhabited area delimited by blue lines. c – Periurban area of Quillacollo city in Cochabamba Department, blue line delimiting the urban area from the sylvatic area. d – Rural locality of Thago Thago in Potosi Department, blue lines delimiting the grouped dwellings. In a, b, and d, green dots indicate specific sites where the wild *T. infestans* were collected; red dots represent specific sites where intra-peridomestic *T. infestans* were collected; the names of each population in black. See also Table 2 for the ecotope origin.

*T. infestans* were collected in three areas (localities) located in the Eastern Andean Cordillera and the individuals were distributed into wild, peridomestic, and intra-peridomestic subsamples defined according to space and ecotopes (Figure 1). In wild environments, the insects were captured with mice bait traps [22], and in intra-peridomestic sites using a manual active search during the day by the research team. The three areas belong to high valleys of the Inter-Andean Dry Forest ecoregion characterized by mild weather, wet summers, and cool dry winters; most rainfall occurs during summer from December through March. The dry season is substantially colder and lasts from April through August. Depending on the altitude, maximal and minimal temperatures vary slightly. 

The first locality is Sapini (16°48′47″S, 67°42′10″W, 1880 m), a small village along the Sapahaqui River in La Paz Department, composed of 30 dwellings where approximately one-third are inhabited or abandoned. At the beginning of the study the team took contact to the authority of the village which has permit to search for triatomines around and in the village. The village is surrounded by high mountains and the lands near the river are used for growing vegetables using an irrigation system. Since 2003 the village has been under entomological vigilance by La Paz Department’s Departmental Health Service, visiting the village every 6 months. They found a persistence of dwelling infestation in peridomestic and/or intradomestic sites, ranging from 3.9% to 25% (2003-2010), and was 9.1% at the beginning 2010. The current bugs, collected during October 2010, were grouped into six subsamples, two from intra-peridomestic sites (piles of stones, indoors) and four from wilderness sites: two of them were captured in the cliffs near the village (around 50–100 m away) and two others farther away (300–500 m) in a field of stones and thorns and a pile of stones.

The second area, Quillacollo (17°25′22.03″S 66°17′30.38″W, 2600 m), is located in Cochabamba department at the urban periphery of Quillacollo city, 10 km from Cochabamba city, in the same valley. This suburb was under entomological vigilance by the local municipality authorities, but insecticide spraying of infested/reinfested dwellings was not applied during the study period due to lack of resources from the municipality. The work was developed in collaboration with the authorities of Quillacollo municipality that has provided staff to guide the research team to collect triatomines in the inhabited part. Four populations were collected in peridomestic structures (piles of river stones and piles of bricks where hens often sleep) belonging to different dwellings located at the periphery of the urbanized area; four others were in the wilderness in rock outcrops. The periphery of the urban area is surrounded by hills where there are rock outcrops always infested with *T. infestans*. All these populations were collected from October 2009 to June 2010.

The third area is a very small village, Thago Thago (18° 0′41.53″S 65°48′32.68″W, 2000 m), about 80 km southeast of Quillacollo in Potosi Department. As in the village of Sapini, the study was done in accordance with local authorities and also the community participation. The village has ten dwellings along a small road bordered by two hills. The dwellings were grouped (no more than 200 m apart), except for two that were set apart from the others, about 300 and 760 m away. The dwellings were treated at least once with insecticide before the study, but the village was not monitored by the health authorities during 2010. Seven populations were collected in peridomestic and/or indoor structures belonging to nine different dwellings, and four in the wilderness in rocky areas in the two hills on either side of the group of dwellings. 

### Microsatellite genotyping

DNA was extracted from two bug legs using the slightly modified CTAB-chloroform method [23]. Briefly, legs were crushed in 200 µl of 2% CTAB (cetyl trimethyl ammonium bromide) and incubated overnight at 37°C after the addition of 30 µl of proteinase K (20 mg/ml). The extraction was then performed as described previously [23]. The final DNA pellet was suspended in 20 µl of distilled water. Seven sets of primers identified for *T. infestans* microsatellite loci (TiA02, TiE02, TiC09, TiF11, TiF03, TiD09, TiE12 [24] were used in the polymerase chain reaction (PCR). The forward primer of each set was the 5′ end labeled with one of the three fluorescent dyes, FAM, NED, or VIC (Applied Biosystems, Villebon sur Yvette, France). PCR amplifications were performed in a 25-µl solution containing the following products at the concentrations of 1.5 mM for MgCl2, 200 µM for each dNTP, 0.4 µM for each primer (forward-labeled), 1 U of Taq polymerase (Quiagen, Courteboeuf, France), 1X Taq Polymerase buffer, and 20–25 ng template DNA. Amplifications were carried out in a Mastercycler (Eppendorf, Hamburg, Germany) under the following conditions: a starting step of 94°C for 5 min, 35 cycles at 94°C for 30 s, 50°C for 30 s, and 72°C for 30 s, and a final step at 72°C for 15 min. Amplified products were visualized after electrophoresis on a 2.5% agarose gel. Electrophoreses of fluorescent labeled PCR products, diluted and denatured in 20 µl of HiDi formamide, were carried out on an ABI PRISM310 Genetic Analyzer (Applied Biosystems, Carlsbad, CA, USA), with Genescan 500 LIZ as the internal size standard. GeneMapper^®^ software (Applied Biosystems, Carlsbad, CA, USA) was used to characterize alleles.

### Data analysis

The significance of genotype association between pairs of loci (linkage disequilibrium) was tested using Fstat 2.9.3.2 software [25]. The *P*-values of the test for all pairs of loci was obtained as follows: genotypes at two loci were associated at random 2100 times, and the log-likelihood G-test statistic was recalculated on the randomized data. The *P*-value was estimated as the proportion of statistics from randomized data sets that are larger or equal to the proportion observed. 

Analysis of genetic variability per loci, per sample, and overall consisted in general descriptive indices estimated with Fstat [26]. Allelic richness (aRich) measured the number of alleles independently of sample size, gene diversity per locus, and the sample used as an unbiased estimator [27], and *F*_IS_ index [28] measured the heterozygote deficit or excess. The *F*_IS_ index was calculated for each sample and the Hardy Weinberg equilibrium was tested within the samples by allele permutations among individuals (1000 times) using Fstat. The 95% confidence interval of the *F*_IS_ values per sample was estimated by bootstrapping the alleles within each sample using the GENETIX v 4.05.2 package [29]. Tests for differences among groups of populations for allelic richness, gene diversity, and *F*_IS_ between localities were carried out using Fstat.

The level of genetic structuring was estimated through different hierarchical analyses of molecular variance (Analysis of Molecular Variance, AMOVA), with Arlequin software v. 3.5.1.2 [30]. The pairwise *F*_ST_ indices can be used as short-term genetic distances between samples and were computed with Arlequin. Differentiation between samples was tested by different types of permutation for each covariance component. The genetic relationships between samples were inferred with the neighbor-joining method (NJ) [31] using Cavalli Sforza’s genetic distances between pairwise samples. This analysis was conducted in Population v. 1.2.30. software (Olivier Langella, CNRS UPR9034; http://bioinformatics.org/~tryphon/populations/). 

A Bayesian approach was implemented to determine the number of genetic clusters within the entire data set using STRUCTURE v. 2.2 [32,33]. Runs with a 50,000-iteration burn-in period and 50,000 iterations in length, up to 100,000 for Thago Thago populations, with variable genetic clusters (k = 2–10) were carried out to identify the best assignment. This procedure places individuals in k clusters chosen in advance and membership coefficients are calculated for each individual in inferred clusters. The “admixture” model for the ancestry of individuals (individuals may have ancestry from various populations) was applied to the data sets with the "Allele Frequencies Correlated" option (meaning that allele frequencies in the different populations are most probably similar), with the other parameters left at their default values. For these runs, the LOCPRIOR option was used, which allows clustering by using sampling locations of individuals as prior information. This option is more appropriate for data sets with few markers and small sample sizes. Five independent runs for each k were produced to assess the consistency of the results across runs, one of the criteria to estimate the best k [32]. Further analysis with STRUCTURE HARVESTED website program (http://taylor0.biology.ucla.edu/structureHarvester/) was applied to visualize likelihood values across the multiple runs performed for the overall data set and the locality data sets and to help determine the best number of clusters corresponding to the data set [34]. 

GENECLASS2 software [35] was used to detect the putative first-generation migrants within the data set in each locality. To test the probability of being a first-generation migrant, the probability for each bug of being a resident (not a migrant) was expressed using Bayesian assignment criteria [36] and the Monte-Carlo resampling method [37]. Also, the probability that the putative population source of the first-generation migrant was among the subsamples within each locality was tested using the assignment-exclusion test implemented in GENECLASS2 with the same model as for the detection of migrants.

## Results

### Genetic variability overall and within each locality

Data from seven previously described microsatellite loci were analyzed for a total of 277 *T. infestans* specimens collected in three localities in wild and domestic environments (Sapini, Quillacollo and Thago Thago). The overall amplification success of the loci was 97.7%, ranging from 92.8% (locus TiD09) to 99.3% (loci TiE02, TiC09, TiE12). All loci were polymorphic. No significant association between the pairs of locus genotypes within the overall sample and within each sampled locality was found at the nominal 1% level, a result supporting the statistical independence of loci. The number of alleles per locus in the entire sample ranged from three (TiF11) to 19 (TiF03), and the mean allelic richness was 8.0 ± 3.9. Genetic variability indices in the three samples composed of all analyzed individuals in each locality are presented in Table 1. Significant deviations from HW expectations due to heterozygote deficit (positive *F*_IS_ values significantly different from random predictions) were observed in Quillacollo and Thago Thago but not in Sapini (Table 1). The locality pairwise comparison showed that Sapini differed for all indices (aRich, observed proportion of heterozygotes, gene diversity, and *F*_IS_) from Quillacollo and Thago Thago (*P* < 0.02), while only the *F*_IS_ value was significantly different between Quillacollo and Thago Thago (*P* < 0.01). Samples from each locality were divided into subsamples according to space and ecotopes (intra-peridomestic or wild) and the summary of genetic variability, per locus and per subsample, is presented in Table 2 for Sapini, Quillacollo, and ThagoThago. Remarkably, in Sapini two loci were monomorphic among all the subsamples (TiE02 and TiF11) and up to six loci in the Dom2 and IP11 subsamples, while in Quillacollo and Thago Thago, out of 19 subsamples, only one was monomorphic at one locus, TiF11 (CV119, Quillacollo). Within the 25 subsamples, no significant deviations from HW expectations were observed except for one subsample in Thago Thago (CV78) composed of bugs collected in a single dwelling (significant positive *F*_IS_). 

**Table 1:** Genetic indices evaluated among seven microsatellite loci per locality, Sapini, Quillacollo and Thago Thago.

Genetic indices	Locality		
	Sapini	Quillacollo	Thago Thago
Av. N ± SD	37.1 ± 1.6	115.6 ± 1.7	116.7 ± 5.0
Av. aRich ± SD	2.13 ± 1.33	5.97 ± 2.58	7.13 ± 3.3
Av. GD ± SD	0.28 ± 0.25	0.69 ± 0.18	0.72 ± 0.14
*F*_IS_	0.042	0.064	0.181
*P*-value for *F*_IS_	0.27	0.0010*	0.0005*

Av., average among loci; SD, standard deviation; N, number of individuals; aRich, allelic richness; GD, genetic diversity; *P*-value for *F*_IS_ was based on 2100 randomisations the nominal level for 5% was 0.00238; * significant value.

**Table 2:** Summary of the genetic variability for seven microsatellite loci in intra-peridomestic and wild subsamples of *T. infestans* in Sapini, Quillacollo and Thago Thago localities.

Subsample	Ecotope	Locus							
		TiA02	TiE02	TiC09	TiF11	TiF03	TiD09	TiE12	Overall loci
									Av. ± SD
Sapini									
Dom1	Intra-peridomestic								
N		6	6	6	4	6	6	6	5.7 ± 0.7
aRich		1.98	1	1.98	1	2.91	2.00	2.00	1.84 ± 0.66
*F*_IS_		-0.250	NA	0.615	NA	0.524	-0.667	-0.667	-0.053
Dom2	Peridomestic								
N		6	6	6	6	5	6	6	5.8 ± 0.4
aRich		1	1	1	1	2.96	1	1	1.28 ± 0.7
*F*_IS_		NA	NA	NA	NA	-0.333	NA	NA	-0.333
Syl1	Wild								
N		7	7	7	6	7	7	7	6.9 ± 0.4
aRich		1.99	1	1	1	3.39	2.00	2.00	1.77 ± 0.87
*F*_IS_		-0.500	NA	NA	NA	-0.263	-0.714	-0.714	-0.525
IP11	Wild								
N		7	7	7	7	7	7	7	7.0 ± 0.0
aRich		1	1	1	1	2.890	1	1	1.27 ± 0.71
*F*_IS_		NA	NA	NA	NA	-0.412	NA	NA	-0.412
Z11	Wild								
N		7	7	7	6	7	7	7	6.8 ± 0.4
aRich		1.98	1	1	1	3.24	1.99	1.99	1.74 ± 0.82
*F*_IS_		-0.333	NA	NA	NA	0.020	0.143	0.143	0.006
Z10	Wild								
N		5	5	5	5	4	5	5	4.8 ± 0.4
aRich		1	1	1	1	3.00	1.98	1.98	1.56 ± 0.78
*F*_IS_		NA	NA	NA	NA	0.294	-0.143	-0.143	0.077
Quillacollo									
CV119	Peridomestic								
N		15	16	16	16	16	16	16	15.8 ± 0.4
aRich		2.975	2.659	2.920	1.000	3.980	3.751	2.779	2.86 ± 0.96
*F*_IS_		0.125	-0.098	0.125	NA	-0.254	0.174	-0.099	0.002
CV125	Peridomestic								
N		9	9	9	8	8	5	7	7.8 ± 1.5
aRich		2.554	2.817	2.817	1.992	2.000	3.000	1.989	2.45± 0.45
*F*_IS_		-0.474	-0.655	-0.067	-0.273	0.000	-0.333	0.625	-0.208
CV134	Peridomestic								
N		11	11	11	11	11	10	11	10.8 ± 0.4
aRich		3.688	3.385	3.026	2.387	5.118	2.747	2.883	3.32 ± 0.90
*F*_IS_		-0.104	-0.184	-0.188	0.059	0.011	0.063	0.101	-0.046
CV59	Peridomestic								
N		18	18	18	18	18	18	18	18.0 ± 0.0
aRich		4.089	2.957	3.287	1.987	5.385	5.100	3.619	3.62 ± 1.19
*F*_IS_		0.215	-0.172	0.182	0.370	0.056	0.004	-0.245	0.039
P1	Wild								
N		15	15	15	15	15	15	15	15.0 ± 0.0
aRich		3.264	2.718	3.766	1.988	5.176	4.261	3.530	3.53 ± 1.03
*F*_IS_		0.131	0.234	0.149	-0.077	-0.130	-0.277	-0.171	-0.026
P2	Wild								
N		14	14	14	14	14	14	14	14.0 ± 0.0
aRich		3.650	2.592	2.899	1.997	5.137	4.181	4.999	3.64 ± 1.21
*F*_IS_		-0.004	-0.020	-0.156	0.409	-0.095	-0.143	0.010	-0.018
P3	Wild								
N		22	22	22	22	22	22	22	22.0 ± 0.0
aRich		3.577	3.437	2.969	1.744	3.943	4.370	4.958	3.57 ± 1.03
*F*_IS_		0.016	-0.112	-0.141	0.784	0.088	-0.050	0.003	0.007
P4	Wild								
N		12	12	12	12	12	12	12	12.0 ± 0.0
aRich		4.214	3.190	2.416	1.417	5.706	5.853	4.028	3.83 ± 1.63
*F*_IS_		0.025	-0.329	-0.197	0.000	0.143	-0.052	-0.111	-0.063
Thago Thago									
CV7687	Intra-peridomestic								
N		13	12	12	13	13	11	13	12.4 ± 0.8
aRich		3.66	3.04	2.41	2.15	3.91	3.73	3.35	3.18 ± 0.68
*F*_IS_		0.032	0.017	0.492	0.231	0.253	-0.029	0.065	0.130
CV85	Peridomestic								
N		4	4	4	4	3	4	4	3.9 ± 0.4
aRich		2.50	2.71	1.75	2.00	2.00	1.96	4.21	2.45 ± 0.85
*F*_IS_		0.500	-0.286	0.000	0.571	-0.333	-0.200	0.455	0.156
SN0102	Intra-peridomestic								
N		17	19	18	18	18	16	19	17.9 ± 1.1
aRich		3.21	2.54	2.36	1.97	3.71	4.02	3.84	3.09 ± 0.81
*F*_IS_		0.074	0.179	0.526	0.452	0.012	-0.047	-0.038	0.121
CV82	Intra-peridomestic								
N		8	8	8	8	8	4	8	7.4 ± 1.5
aRich		2.94	2.95	1.62	1.97	3.99	4.39	3.00	2.98 ± 0.99
*F*_IS_		0.646	0.041	-0.077	0.517	0.263	0.182	-0.029	0.247
CV74	Intra-peridomestic								
N		10	10	10	10	9	8	10	9.6 ± 0.8
aRich		3.35	3.02	3.02	1.96	3.90	4.30	3.43	3.28 ± 0.75
*F*_IS_		0.087	0.211	0.211	0.386	-0.094	-0.167	0.031	0.066
CV78	Intra-peridomestic								
N		13	13	13	12	13	13	13	12.9 ± 0.4
aRich		3.52	3.14	3.47	1.71	3.85	4.21	3.15	3.29 ± 0.79
*F*_IS_		-0.114	0.439	0.306	0.436	0.446	0.284	0.208	0.274*
CV68	Peridomestic								
N		6	6	6	6	5	6	6	5.8 ± 0.4
aRich		3.04	2.82	1.50	1.99	4.50	3.81	3.23	2.98 ± 1.02
*F*_IS_		-0.026	-0.250	0.000	0.706	0.111	0.184	-0.136	0.081
C11	Wild								
N		12	15	15	14	14	13	15	14.0 ± 1.1
aRich		3.48	2.18	2.69	1.95	4.23	3.68	3.86	3.15 ± 0.88
*F*_IS_		0.107	0.279	0.590	0.103	0.259	-0.082	0.099	0.186
C12	Wild								
N		15	14	15	14	14	14	15	14.4 ± 0.5
aRich		2.77	2.48	3.14	2.11	3.69	3.76	3.78	3.10 ± 0.67
*F*_IS_		-0.116	0.027	0.357	0.406	-0.273	-0.087	-0.087	0.010
C13	Wild								
N		12	14	14	14	13	13	14	13.4 ± 0.8
aRich		3.61	1.64	2.55	2.76	3.47	4.47	3.80	3.18 ± 0.94
*F*_IS_		-0.063	-0.040	0.090	0.266	-0.109	0.040	-0.068	0.015
C2	Wild								
N		5	5	5	5	5	5	5	5.0 ± 0.0
aRich		3.07	3.93	2.00	1.97	3.43	4.17	4.50	3.29 ± 1.01
*F*_IS_		0.077	0.294	0.667	0.600	0.250	-0.176	-0.143	0.180


N, number of individuals; aRich, allelic richness; Av., average among loci; SD, standard deviation; *P*-value for *F*_IS_ was based on 4200 randomizations, the nominal level for 5% was 0.00119, there was no significant value; NA, not available.

### Genetic structure and differentiation between intra-peridomestic and wild populations

**Figure 2:**
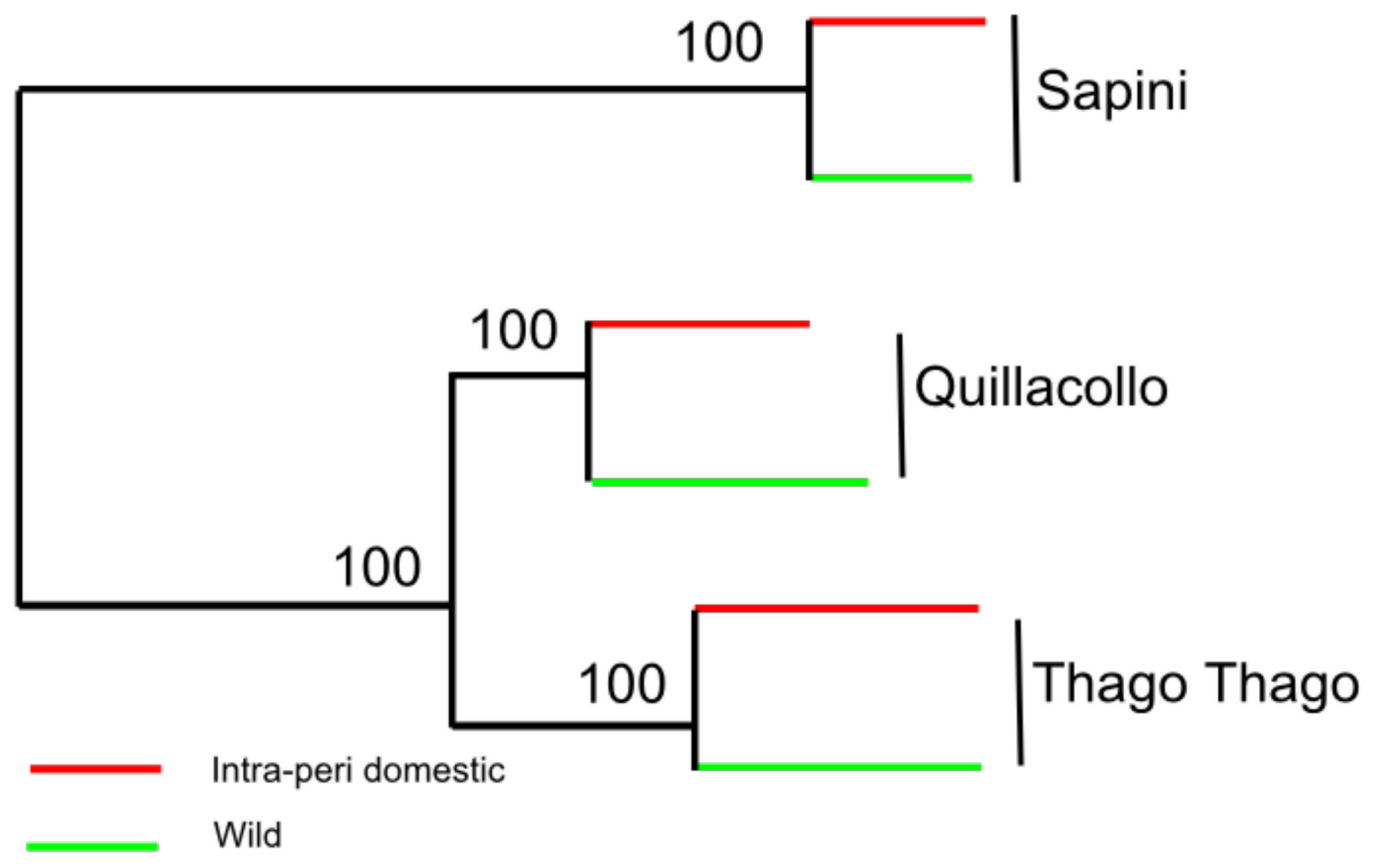
Neighbor-joining between wild and intra-peridomestic populations. The tree was built from Cavalli Sforza’s genetic distances between the six populations that grouped, in each locality (bugs collected in the wild and the intra-peridomestic environments). The bootstrap values are expressed at the nodes of the branches.

The genetic structure of the subsamples clustered according to different spatial levels was explored with the analysis of molecular variance (AMOVA) using all loci for distance computation and all data, with 0.12% threshold for missing data (Table 3). Among the 25 subsamples, most of the genetic variation was assigned within individual (78.7% of the total variation, *P* < 10^−4^, see Table 3), but a significant 19.5% variation was attributed to differences between subsamples, showing some genetic differentiation among them (*P* < 10^−4^). The first hierarchical AMOVA, grouping subsamples according to their geographic origin (locality), showed a higher variance component among localities (18%) than among subsamples within localities (6.7%), and in both cases the *F*_CT_ (among localities) and *F*_SC_ (among subsamples within localities) indices were significant (*P* < 10^−4^), showing geographical structuring between localities and also some possible structuring within localities. This geographical structure was fully confirmed by the topology of the neighbor-joining tree built from Cavalli Sforza’s genetic distances between wild and intra-peridomestic populations from the three localities, where the bootstrap values at the nodes grouping each locality were highly significant (Figure 2). To further explore a possible structure between ecotopes (wild/intra-peridomestic), additional hierarchical AMOVA were performed within each locality. In the three localities, the variability between the two kinds of ecotope accounted for a very low total variability rate, 0.30% in Quillacollo, 1.48% in Sapini, and 1.66% in Thago Thago, and the differentiation between the two ecotopes was not significant for Sapini and Quillacollo and borderline for Thago Thago (Table 3). However, in each locality the variance component attributed to differences between subsamples (hence among wild or domestic groups of subsamples) was significant, showing some genetic differentiation between subsamples within ecotopes. Moreover, pairwise comparisons between subsamples using the *F*_ST_ estimator showed significant differentiation between most of the subsamples (94% of the 300 pairwise comparisons, data not shown). 

**Table 3:** Analysis of Molecular Variance (AMOVA) for *T. infestans* samples according to different hierarchical levels.

Hierarchical level	Source of variation	d. f.	% of variation	*F*-index	*P*-value*
Subsamples (25)	Among subsamples	24	19.50	*F*_ST_ = 0.195	< 10^-4^
	Among individuals within subsamples	252	1.82	*F*_IS_ = 0.022	0.15
	Within individuals	277	78.70	*F*_IT_ = 0.213	< 10^-4^
Localities (Sapini, Quillacollo, Thago Thago)	Among localities	2	17.98	*F*_CT_ = 0.180	< 10^-4^
	Among subsamples within localities	22	6.74	*F*_SC_ = 0.082	< 10^-4^
	Among individuals within subsamples	252	1.70	*F*_IS_ = 0.023	0.15
	Within individuals	277	73.59	*F*_IT_ = 0.264	< 10^-4^
Ecotopes in Sapini (wild / peri-intra domestic)	Among ecotopes	1	1.48	*F*_CT_ = 0.015	0.40
	Among subsamples within ecotopes	4	21.46	*F*_SC_ = 0.218	< 10^-4^
	Among individuals within subsamples	32	-16.81	*F*_IS_ = -0.218	0.98
	Within individuals	38	93.86	*F*_IT_ = 0.061	0.42
Ecotopes in Quillacollo (wild / peridomestic)	Among ecotopes	1	0.30	*F*_CT_ = 0.003	0.31
	Among subsamples within ecotopes	6	8.59	*F*_SC_ = 0.086	< 10^-4^
	Among individuals within subsamples	109	-2.57	*F*_IS_ = -0.028	0.87
	Within individuals	117	93.69	*F*_IT_ = 0.063	0.01
Ecotopes in Thago Thago (wild / peri-intra domestic)	Among ecotopes	1	1.66	*F*_CT_ = 0.017	0.048
	Among subsamples within ecotopes	9	5.61	*F*_SC_ = 0.057	< 10^-4^
	Among individuals within subsamples	111	11.82	*F*_IS_ = 0.127	< 10^-4^
	Within individuals	122	80.91	*F*_IT_ = 0.190	< 10^-4^

d.f., degree of freedom; * *P*-value for *F*-index based on 10,000 permutations.

**Figure 3:**
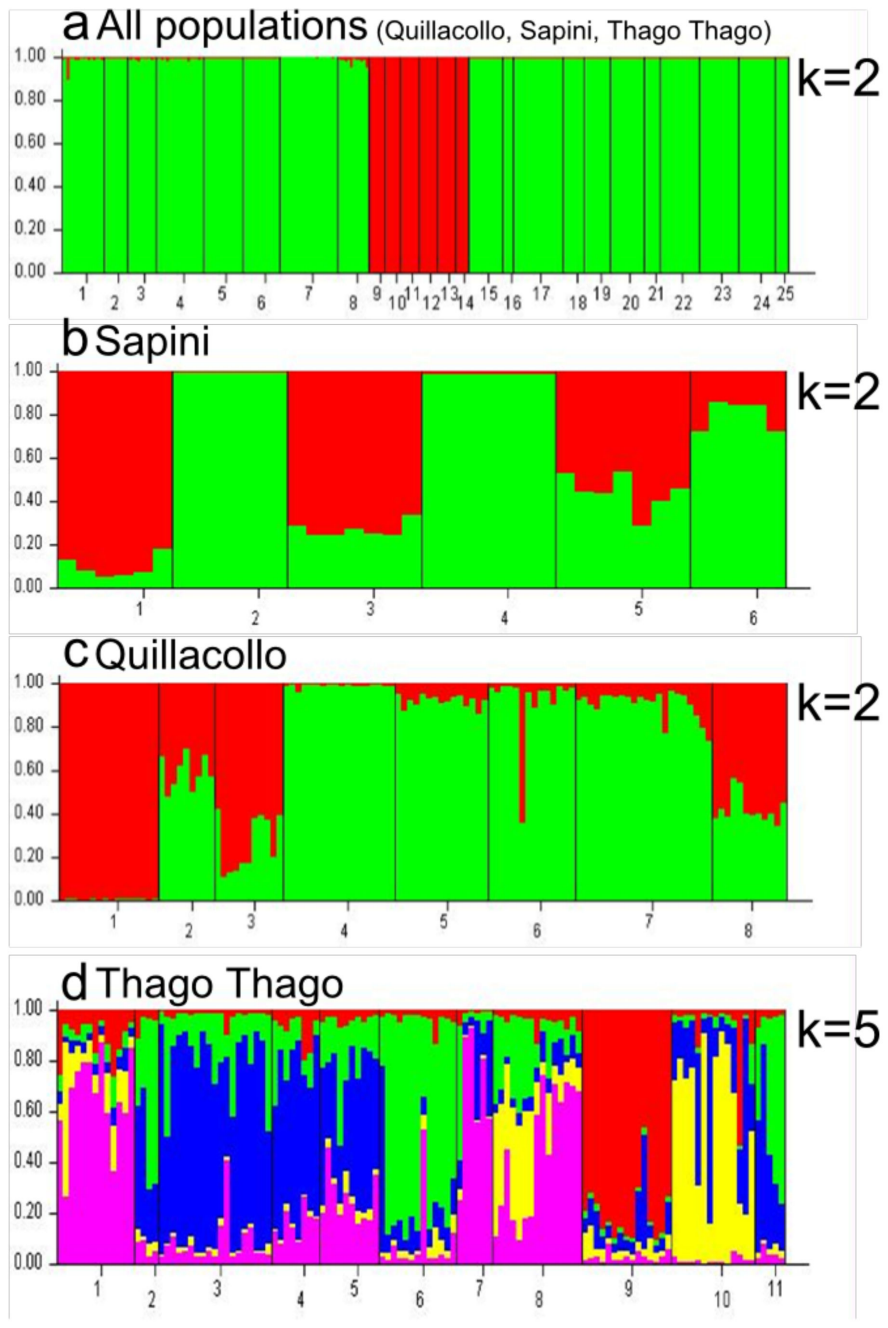
Plots of ancestry estimates of *T. infestans* individuals using STRUCTURE software. a – Plot of the individuals belonging to the 25 study populations, one to four intra-peridomestic populations from Quillacollo, five to eight wild populations from Quillacollo, nine and ten intra-peridomestic populations from Sapini, 11 to 14 wild populations from Sapini, 15 to 22 intra-peridomestic populations from Thago Thago, 23 to 25 wild populations from Thago Thago. b - Plot of the individuals belonging to the six populations in Sapini (one and two intra-peridomestic, three to six wild). c - Plot of the individuals belonging to the eight populations in Quillacollo (one to four intra-peridomestic, five to eight wild). d - Plot of the individuals belonging to the 11 populations in Thago Thago (one to eight intra-peridomestic, nine to 11 wild). Each individual in the data set is represented in the plots by a single vertical line, which is partitioned into different colored segments representing the individual membership estimates in each of the inferred clusters (k).

The Bayesian method of the assignment implemented in STRUCTURE detected two clusters in the overall sample (Figure 3a) whether the “admixture” or the “no admixture” model was applied. One genetic cluster was composed of all the Sapini subsamples and the second one all the Quillacollo and Thago Thago samples, indicating a clear geographical differentiation. To explore the population structure in each locality in greater detail, the corresponding subsamples were analyzed separately. In Sapini and Quillacollo, two genetic clusters were detected (Figure 3b and c), and no correlation was observed with the bugs’ capture area, intra-peridomestic vs. wild. The individual patterns of assignment to the two genetic clusters were generally homogeneous within subsamples that were defined based on spatial criteria (grouping collected bugs in the same place or nearby). This pattern may depict the existence of discrete populations, in domestic as well as in wild ecotopes. Similar individual genetic assignments in wild and intra-peridomestic subsamples were also observed in Sapini and Quillacollo: some subsamples were composed of members assigned to a single cluster, while others were composed of individuals that presented an admixed origin. In Thago Thago, there were five significant clusters (Figure 3d, Table 4). Only 50% of the individuals were assigned to one of the five inferred clusters, with a probability greater than 70%. The other individuals came from two or three clusters. Only two populations contained the majority of its individuals assigned to a single cluster (C12 wild and CV78 intra-peridomestic). 

**Table 4:** Assignement of individual genotypes of *T. infestans* belonging to 11 subamples of Thago Thago to five inferred clusters.

Subsample	No. of *T. infestans*	Number of individuals assigned to each inferred cluster with a probability > 0.70					No assigned
		Five inferred clusters (k = 5)					
		1	2	3	4	5	
CV7687	13	0	0	0	0	5	8
CV85	4	0	0	0	0	0	4
SN0102	19	0	0	12	0	0	7
CV82	8	0	0	3	0	0	5
CV74	10	0	0	0	0	0	10
CV78	13	0	9	1	0	0	3
CV68	6	0	0	0	0	3	3
C11	15	0	0	0	0	4	11
C12	15	13	0	0	0	0	2
C13	14	0	0	2	7	0	5
C2	5	0	1	1	0	0	3

### Detection of putative first-generation migrants within localities

In each locality, the putative first-generation migrants were detected on the basis of the highest likelihood ratio calculated between the subsample from which the individual was sampled and among all other subsamples in the locality including its own subsample (Table 5). Putative first-generation migrants were detected in 12 of 25 samples, the migrant rates were 5.3% (two migrants) in Sapini, 2.6% (three migrants) in Quillacollo, and 7.4% (nine migrants) in Thago Thago. Of the 14 putative migrants, four were males, four females, and six third-, fourth-, and fifth-stage nymphs. Eight were captured in intra-peridomicile and seven in wild ecotopes, six could have originated from one or several other subsamples of the corresponding locality (the assignment-exclusion test in GENECLASS software provided a significant probability P_2_ > 0.70, Table 5). The results supported the displacement of bugs from wild to intra-peridomestic ecotopes in Sapini and Thago Thago, and also displacements between intra-peridomestic ecotopes in Thago Thago. In Quillacollo, the origin of the migrant remained unknown.

**Table 5:** Putative first generation migrants and possible origin.

*T. infestans* code	Locality	Stage	Population where the individual was sampled			Putative original population			Distance between populations (m)	Putative ecotope displacement**
			Ecotope	Name	*P_1_*	Ecotope	Name	*P_2_*		
Sap 398	Sapini	Female	Intra-peri	Dom1	< 0.05	Wild	Syl1	0.95	65	Wild to domestic
Sap 471	Sapini	5^th^ nymph	Wild	Z10	< 0.05	Wild	Z11	0.76	320	Wild to wild
Qui 253	Quillacollo	4^th^ nymph	Peri	CV125	< 0.01	Wild	P1	0.21	250	uk
Qui 379	Quillacollo	4^th^ nymph	Peri	CV59	< 0.01	Wild	P1	0.27	175	uk
Qui 659	Quillacollo	Male	Wild	P2	< 0.001	Wild	P3	0.45	233	uk
Tor 622	Thago Thago	5^th^ nymph	Intra-peri	CV7687	< 0.01	Wild	C13	< 0.01	150	uk
Tor 369	Thago Thago	Female	Intra-peri	SN0102	< 0.001	Intra-peri	CV78	< 0.006	120	uk
Tor 404	Thago Thago	Female	Intra-peri	CV82	< 0.01	Wild	C12	0.72	125	Wild to domestic
Tor 149	Thago Thago	Male	Intra-peri	CV78	< 0.001	Intra-peri	CV82 or SN0102*	0.86	130	Domestic to domestic
Tor 246	Thago Thago	Male	Peri	CV68	< 0.001	Wild	C11	0.42	1000	uk
Tor 494	Thago Thago	Male	wild	C11	< 0.01	Intra-peri	CV7687	0.39	40	uk
Tor 435	Thago Thago	1^st^ nymph	Intra-peri	CV7687	< 0.01	Wild	C13	0.49	150	uk
Tor 442	Thago Thago	Female	Wild	C13	< 0.001	Wild	C12	0.84	140	Wild to wild
Tor 502	Thago Thago	4^th^ nymph	Wild	C13	< 0.01	Wild	C2	0.80	200	Wild to wild

P_1_ is the probability to be a resident that is to say to be no a first generation migrant; P_2_ is the probability to originate from the putative population detected by the test; * both subsamples had the same probalility; ** domestic includes peri or intra domestic origins, putative origin was considered when P_2_ was > 0.70; uk, unknown; GENECLASS software was applied for these analysis.

## Discussion

### Genetic diversity in *T. infestans*

The overall genetic diversity of *T. infestans* observed in Quillacollo and Thago Thago is high and similar to that previously observed in the same area analyzing sylvatic populations with the same set of microsatellite loci [38]. Strong genetic variability has also been found in several Argentinean populations, each composed of insects from several houses in the same village [39] and even at the micro-geographical scale among house compound populations [16]. Therefore, Sapini seems to be an exception with significantly much lower genetic diversity than in Quillacollo and Thago Thago (0.28 vs. around 0.70). In Sapini two loci were monomorphic, TiF11 and TiE02, analyzed for 34 and 38 insects, respectively. Low genetic variability was also previously detected in Sapini upon analysis of the polymorphism of cytochrome b sequences; indeed, a single haplotype belonging to a “special group of haplotypes” composed of very divergent haplotypes was identified [40]. This group of divergent haplotypes was sampled only in northern Andean valleys of La Paz Department, but not in the Cochabamba region. The authors suggested the intervention of isolation and bottleneck phenomena due to the particular feature of these valleys, highly fragmented, with extreme weather conditions and where many endemic species have evolved [21]. The low genetic diversity currently observed for microsatellite polymorphisms in these populations is in agreement with this hypothesis.

### Panmictic unit

The search for the size of the natural panmictic unit contributes to determining the geographical scale where random mating among members of a species is potentially possible. The putative panmictic unit is the most suitable to define populations for population genetics analysis to derive the relationships that exist between the members of these populations at different geographical scales, which is needed for control strategies. The allozyme polymorphism analyses first showed that the *T. infestans* members within a village evolve in agreement with the assumption of panmixia [41]; then it was proposed that the panmictic unit can be smaller, such as populations limited to individual houses or peridomestic structures in a village [20]. Other studies with microsatellite markers generally agree with this last assumption for *T. infestans* [16,39,41] as well as for other species of triatomine such as *Triatoma longipennis* for which the panmictic unit corresponds to a collection of insects captured in a single peridomestic structure [42]. However, for *Triatoma dimidiata* from Guatemalan populations, the results suggest that the size of the panmictic unit is not the same in different regions, probably due to variable migration rates between populations [43]. Here, Hardy-Weinberg equilibrium was observed within all populations studied except one. The above-mentioned overall results show that, to develop genetic studies of triatomine populations, the best way is to consider a group of insects collected in a single capture site or sites that are extremely close (a few meters) in the same period, as a population. This approach better assesses the genetic relationships between populations in each environment and can infer the dispersal ability of insects in a given area, a frequently raised question that is directly related to the epidemiology of the disease.

### Genetic structure in *T. infestans*

In the present case, a strong geographic structure was observed between the three localities (the *F*_ST_ value is 0.179, P < 10^-5^), up to 242 km away (Sapini to Thago Thago) as observed between populations collected in different localities in Argentina over long distances [44]. However, high genetic structure was also depicted at the locality scale among wild and intra-peridomestic populations. It is increasingly common and expected to see strong structuring among populations from peridomestic collection sites because the bugs may be less inclined to disperse in this medium than in sylvatic areas, since food host mammals are locally more stable. Few data exist concerning wild sites. The previous data regarding wild populations collected near Quillacollo, whose maximal distance is about 1 km, showed differentiation between only some of them [38], while in the current analyses of the four wild populations of Quillacollo, most of the pairwise *F*_ST_ value comparisons showed a significant differentiation (of 18 comparisons, 14 cases were significant). These results also support restricted gene flow among wild populations at a small geographic scale that can be explained by the conjuncture of two phenomena: an intrinsically low ability of *T. infestans* to move actively by walking or flying in the case of adults and the foundation of colonies mostly created by few founder specimens (founder effect).

Even in this context, the dispersion remains an important parameter that leads to major epidemiological consequences, such as the colonization of new ecotopes. The factors influencing dispersion are still poorly known. In addition to the physical parameters, the search for blood meals is mandatory for triatomine survival. This feeding habit links the insects to their guests, whose dynamics, whatever the medium, is strongly dependent on the level of anthropization. The environmental conditions and their changes may influence the dispersal patterns, which in fact could be very different depending on the environmental pressures.

### Genetic flow of *T. infestans* between wilderness and human habitat

The analysis of genetic flow between *T. infestans* collected in wild or domestic areas is a very new issue in understanding reinfestion events in areas treated by insecticide spraying. Indeed, *T. infestans* has long been considered exclusively domestic except in the valleys of Cochabamba, Bolivia; therefore, the problem was geographically limited and not fully treated. This is not the case of the vector species, which plays an epidemiological role in other endemic areas and whose wild populations are recognized as threatening because of their dispersion to inhabited areas [45-47]. 

In the case of *T. infestans*, the first work of morphometry applied to domestic and wild populations from the Cochabamba valley highlighted differences that have been interpreted as incipient separation [48] because they had not been observed by enzymatic analysis before [49]. Moreover, in this same area, the comparison of reinfesting populations with domestic populations before treatment and the surrounding wild populations, using the morphometric marker, have led to the conclusion that reinfesting specimens came from residual populations [50]. In contrast, other analyses, based on mitochondrial sequence comparison, showed shared haplotypes between wild and domestic bugs, indicating a common origin [5,17,40,51]. In addition, major variability was observed in the wild environment, one argument in favor of the origin of dispersion from the wild to human habitats [40]. At a small geographic scale, microsatellite markers appeared to be more informative of bug movement between populations of different ecotopes, but few data are currently available for *T. infestans*. In Argentina, a microsatellite polymorphism study argues for genetic differentiation between a wild and a peridomestic population, 7 km apart [51], while a significant link between two sylvatic foci and the nearest domestic/peridomestic bug populations (100 m and 1.2 km away) was found in an Argentinean area with recurrent reinfestation, raising the hypothesis of sylvatic contributors to reinfestation [5]. 

In the current study, the analytical effort to estimate gene flow between different ecotopes is based on (1) the sampling strategy that grouped the bugs in potentially panmictic populations using the spatial criterion and (2) the use of microsatellites, which are highly polymorphic markers. 

The absence of significant differentiation between the insects collected in intra-peridomestic and nearby wild environments favors the hypothesis of bug dispersion between ecotopes and the putative sylvatic origin of the reinfesting bugs. The analyses conducted with the STRUCTURE program confirmed this result, showing that close intra-peridomestic and wild populations may be composed of members that are genetically very similar.

### Epidemiological implications of dispersing from wilderness to human habitat

The first study by [5] and the present one strongly suggest that wild populations of *T. infestans* can reach intra-peridomestic areas. For *T. infestans*, it seems that this flow of insects between the two environments has previously existed in different areas because a higher level of genetic relatedness exists between wild and intra-peridomestic in each geographical area than between areas [40]. This process could originate from multiple geographical domestication events, more likely than the occurrence of a single event, in the Bolivian Andes, followed by a recent expansion using passive transport associated with human migrations [13,52,53]. The consequences are the recurrent arrival of new bugs after villages have been sprayed with insecticide and recolonization primarily of peridomestic areas [54]. To date, there is no data showing specific properties of wild populations that can be related to a lower adaptive capacity to the intra-peridomestic environment compared to the domestic environment. Remarkably, we have shown that these wild populations, discovered in abundance in the Andean valleys of Bolivia, also feed on humans when the latter conduct outdoor activities [55]. More generally, the feeding behavior of numerous triatomine species is eclectic, based on a wide range of mammal species blood meal sources. Interestingly, they can also feed on birds and even reptiles. Several studies have shown that triatomine species sporadically found indoors (incursive species) easily feed on humans when they enter dwellings [56,57]. It now seems clear that the wild populations of *T. infestans* represent a health danger, but few data are available on the kinetics of the recolonization process of the human habitat by these populations.

The Southern Cone Initiative to Control/Eliminate Chagas (INCOSUR, http://www1.paho.org/english/hcp/hct/dch/incosur.htm. Accessed 2013 Oct 21), launched in 1991 to control *T. infestans*, has met extensive success in Brazil, Chile, Paraguay, and Uruguay, but control failures have been declared in the Gran Chaco of Argentina, Bolivia, and Paraguay [17,58,59]. In accordance with the objectives of the program, transmission interruption has been officially declared, region by region, wherever mean dwelling infestation rates below 3% are reported. In 2011, transmission interruption was achieved in La Paz Department. This certification was based on the indisputable effectiveness of the work carried out for several years in this department where the rate of dwelling infestation in the endemic municipalities concerned has decreased from 40% to less than 1% (http://www.paho.org/, Southern Cone Initiative to Control/Eliminate Chagas Disease (INCOSUR), Annual meeting 2011). The certification process is a tool that validates the efforts made and the effectiveness of the eradication actions, but monitoring by health authorities often breaks down thereafter, and these attitudes should be precisely examined to prevent transmission risk from persisting especially in areas (Bolivian Andes) where population flows between wild and domestic environments are strongly suspected. This issue was recently fully addressed by emphasizing the role of native vector populations, which occur in natural ecotopes and reinvade/reinfest human habitats [60]. Native populations can be composed of members of the same species as those combatted in the intra-peridomestic environment or by other species. In fact, endemic regions where triatomines do not exist in the wilderness are probably uncommon. Also, public monitoring of Chagas disease should be maintained. This inventory, most particularly the discovery of a far wider distribution of wild *T. infestans* populations than initially described, requires further research to understand the dynamics of wild population movements and their ability to adapt to new ecotopes. In monitoring, emphasis on inhabitant-based participation rather than the systematic search for triatomines by professional teams, which is very costly, could be considered by the health authorities, as well as prioritizing awareness and education campaigns for the human populations at risk [57,61]. Sustained action over time is therefore the major challenge to fight against this new paradigm of Chagas disease. 

It would be incomplete not to mention that the importance of the persistence of dwelling infestation by *T. infestans* in the Gran Chaco, is more likely related to other phenomena than reinfestation by wild populations; one of the involved mechanisms is the development of a pyrethroid resistance [62-64]; however, other factors such as the reduced effectiveness of pyrethroids in peridomestic environment (lack of insecticide stability caused by climatic conditions, intricate peridomestic structures) or the incomplete covering of the village leaving uncontrolled/untreated dwellings, may play a role [11]. In fact, these other phenomena also need to be explored in any area where the reinfestation persists. 
